# Vascular Caruncle of the Urethra

**Published:** 1900-03

**Authors:** A. E. Aust Lawrence

**Affiliations:** Consulting Physician-Accoucheur to the Bristol General Hospital


### VASCULAR CARUNCLE OF THE URETHRA.

BY

A. E. Aust Lawrence, M.D.,

*Consulting Physician-Accoucheur to the Bristol General Hospital.*

[Read before the Bristol Medico-Chirurgical Society, January 10th, 1900.]

Having met with a good number of cases of vascular caruncle of the female urethra, I have thought it well to bring the subject before you in a brief yet practical manner, and I introduce it the more readily as I find that it is still the plan in some books to advise the use of caustics and cautery; and even in those books which advise excision we find that the cautery is recommended to be used after, and a good deal of stress is laid on the danger of bleeding. This I regard as unimportant in the operation I advise and have practised for years both in hospital and private cases. Again, we are told that there is a great liability to recurrence: this I have not found to be the case when complete excision has been practised.

The usual situation of the urethral caruncle is at the orifice of the meatus, and it is generally of small size. I have, however, removed one quite one inch in length, and sessile on the upper urethral wall. It must be borne in mind that some growths are absolutely inside the urethra, and can only be seen when that passage has been dilated; so if, with the usual symptoms of the growth present, nothing can be seen outside, it is well to explore the urethra itself, and most likely about half an inch inside the canal the growth will be detected. The cases I have seen have presented various symptoms, but for all practical purposes they may be divided under three heads: 1. The only symptom complained of was a bearing-down pain and backache, a symptom which is purely pelvic and not likely to draw attention to the urethra, and yet in this case the real cause was a medium-sized growth at the meatus. Removal of the growth removed all the symptoms. There was only a little pain on touching the growth. 2. The only symptom complained of was a constant desire to pass water, and the act of urination was painless. Here complete removal of a small caruncle just inside the urethra cured the patient; the growth was very slightly sensitive. 3. Many cases come under this heading, presenting the usual symptoms of great pain in the act of micturition, also pain in coitus and in standing or walking. The intensity of the symptoms varied in several cases, and the most severe cases were not always those where the growth was largest.

I have operated on cases where these symptoms had existed in a marked degree for many years; one case in the Hospital had a history for eleven years. Many cases that I have operated on had been treated by others with caustics, the actual cautery, and partial excision—in fact, nothing more than snipping off the growth. This is of no use.

The operation I advise is to completely remove the growth. If the whole of the growth can be seen readily by simply stretching the urethral orifice with a pair of sinus forceps, well and good; but if not, then cut the lower part of the meatus and wall of the urethra with scissors, so that you can see the whole of the growth. This cut surface can easily be sewn together with catgut after the growth has been removed. To remove the growth, all you have to do is to take one or two pairs of the old-fashioned pointed artery forceps and catch hold of the urethral mucous membrane beyond the growth. Cut out this portion of the mucous membrane with the growth attached, then bring the edges of the wound together with fine catgut, and you will get perfect union and complete relief of all symptoms, and almost the very first act of micturition will be devoid of pain.

The results of this operation have been in my hands, and in the hands of several medical friends, almost uniformly successful, and where this result has not been obtained it has been due to a bit of the growth being overlooked, and a second more complete operation has cured the patient.

